# In vivo mutagenicity assessment of styrene in MutaMouse liver and lung

**DOI:** 10.1186/s41021-023-00270-9

**Published:** 2023-04-11

**Authors:** Yasumasa Murata, Masakatsu Natsume, Takako Iso, Yoshiyuki Shigeta, Nozomu Hirose, Takaaki Umano, Katsuyoshi Horibata, Kei-ichi Sugiyama, Kenichi Masumura, Akihiko Hirose, Mariko Matsumoto

**Affiliations:** 1 Division of Risk Assessment, National Institute of Health Sciences, Kanagawa, Japan; 2 Genotoxicology Laboratory, BioSafety Research Center Inc, Shizuoka, Japan; 3 Division of Genetics and Mutagenesis, National Institute of Health Sciences, Kanagawa, Japan; 4 Chemicals Evaluation and Research Institute, Tokyo, Japan

**Keywords:** TG488, MutaMouse, Styrene, In vivo mutagenicity, And transgenic rodent gene mutation assay

## Abstract

**Background:**

Styrene (CAS 100-42-5) is widely used as polystyrene and acrylonitrile–butadiene–styrene resin such as plastic, rubber, and paint. One of the primary uses of styrene is food utensils and containers, but a small amount of styrene transferred into food can be ingested by eating. Styrene is metabolized into styrene 7,8-oxide (SO). SO is mutagenic in bacteria and mouse lymphoma assays. It is clastogenic in cultured mammalian cells. However, styrene and SO are not clastogenic/aneugenic in rodents, and no rodent in vivo gene mutation studies were identified.

**Methods:**

To investigate the mutagenicity of orally administered styrene, we used the transgenic rodent gene mutation assay to perform an in vivo mutagenicity test (OECD TG488). The transgenic MutaMouse was given styrene orally at doses of 0 (corn oil; negative control), 75, 150, and 300 mg/kg/day for 28 days, and mutant frequencies (MFs) were determined using the lacZ assay in the liver and lung (five male mice/group).

**Results:**

There were no significant differences in the MFs of the liver and lung up to 300 mg/kg/day (close to maximum tolerable dose (MTD)), when one animal with extremely high MFs that were attributed to an incidental clonal mutation was omitted. Positive and negative controls produced the expected results.

**Conclusions:**

These findings show that styrene is not mutagenic in the liver and lung of MutaMouse under this experimental condition.

**Supplementary Information:**

The online version contains supplementary material available at 10.1186/s41021-023-00270-9.

## Introduction

Styrene (CAS No. 100-42-5) is broadly distributed for commercial use, because of its excellent treatability, low cost, and wide applications. The styrene is industrially synthesized by the dehydrogenation catalysis of ethylbenzene [1]. Mainly, it is used as a raw material for polystyrene and ABS resin such as plastic, rubber, paint and is also used in paint resin, ion exchange resin, and cosmetic ingredients. Polystyrene first became industrialized in 1935. Nowadays, it is pointed out to be one of the most generally known plastic materials. Well-known characteristics of polystyrene are lightness, rigidness, insulation, weather resistance, and transparency. Many styrene consumer products were widely used in every life scene, and one of its main uses is a material of food utensils and containers, as is notified as an additive for styrene resins on a positive list of Japan’s “Food Sanitation Act.” If the food container was made of styrene-based material, a small amount of styrene [2] and styrene oligomers [3, 4] transferred into the food can be consumed. The estimated average amount of styrene was reported as 91.53 ± 26.18 µg/kg in food matrix [2].

Styrene has been researched and verified for a long time due to the numerous exposure opportunities mentioned above. Endogenous styrene metabolism in animals, including humans, has been studied for decades. Styrene is rapidly absorbed in the respiratory and digestive systems and is distributed throughout the body [5–9]. Particularly, these are accumulated mostly in adipose tissues. In the main pathway, styrene is metabolized to styrene 7,8-oxide (SO) by the cytochrome P450 and then, hydrolyzed to the styrene glycol. The styrene glycol is metabolized into mandelic acid and also into phenyl glyoxylic acid. In humans, it was reported that almost all the absorbed styrene is excreted in urine [5, 7]. According to reports, no significant proteins bind styrene and its metabolite, mandelic acid. However, it has been well studied and reported that SO reacts with proteins within the body, implying that it may be the cause of the high reactivity and toxicity [10].

Furthermore, for decades, many investigations and discussions have been conducted in the risk assessment of styrene toxicity. Evaluating mutagenicity is one of the most relevant indications for chemicals to cause genetic damages. i.e. mutation assays, e.g., bacterial reverse mutation (Ames) test and mammalian cell gene mutation assays, are generally applied for the inspection of mutagenicity in vitro. In the previous reports, there are a very large number of positive Ames test results in SO and styrene studies [11–27]. However, the method and the details of their contents were inconsistent among these reports, so the most of these previous results cannot be definitive [10]. The conclusion about the Ames test is equivocal for styrene. However, it was clearly concluded that the metabolite, SO, is mutagenic in vitro, in the Ames test. In the mouse lymphoma assay (MLA), there is no appropriate result for evaluating the mutagenicity of styrene. In just one previous report, which is not OECD TG490 compliant, SO showed clearly positive without S9, although it was negative with S9 [28]. Based on these results, it was concluded that SO without S9 was mutagenic in the MLA. In the hprt assays, for the most results, SO increases the mutant frequency in the hprt gene [23, 29–36]. However, there is little detail of the method in the description. The number of cells counted, the assay difference from the current TG 476, or the descriptive material itself could not be accurately interpreted [10]. As a result, previous in vitro hprt assay results could not be used to assess the mutagenicity of styrene and SO.

The chromosomal aberration (CA) and micronucleus (MN) tests assessing the chromosomal damage were also conducted to evaluation of styrene genotoxicity in 1970s and 1980s. However, the situation is similar to the case of the gene mutation test. Almost all the results were not appropriate for inquiring into the practical baneful effect since these investigations were implemented before the revision and settlement of the OECD TGs. The CA tests of styrene and SO in cultured mammalian cells were investigated [37–45]. Only Jantunen et al. (1986) showed appropriate data to check the clastogenicity [40]. The styrene resulted in clear dose-dependent positive responses with S9 and weak positive responses without S9. About the in vivo rodent CA tests, the investigation of styrene using oral exposure was evaluated [46]. This study found a negative result and suggested that styrene metabolises quickly. It was determined that repeated oral administration of styrene does not induce CAs in rodents in vivo.

Recently, the International Agency for Research on Cancer (IARC) reclassified the carcinogenicity classification of styrene to “Group 2A” (probably carcinogenic to humans, based on limited evidence in human and sufficient in experimental animals) from “Group 2B” (possibly carcinogenic to humans, based on the limited evidence in both humans and rodents) [47]. A carcinogenicity test of styrene was conducted using laboratory mouse and rats. In the B6C3F1 mice, the US National Cancer Institute/National Toxicology Program (NTP) (1979) reported increasing of lung tumorigenesis by gavage exposure at 300 mg/kg /day (78 weeks; 5 days/week) [48]. Ponomarkov and Tomatis (1978) also demonstrated the possible tumor effects of oral administration of styrene in O20 mice [49]. Furthermore, Cruzan et al. (2001) demonstrated that 98–104 weeks (6 h/day; 5 day/week) inhalation exposure causes a significant incidence of lung tumors in CD-1 mice [50]. In the rat, no tumor incidences increased by styrene gavage [48, 51, 52]. Furthermore, the administration in drinking water had no effect on the incidence [53]. Only the inhalation exposure could induce mammary gland tumor and pneumonia [51]. Because styrene exposure induced the lung tumours in mice but not rats, the mechanism of tumor induction has left open for further investigation. Despite the fact that the interpretation of the results and the mechanism of action were ambiguous, there are numerous previous facts indicating the carcinogenicity of styrene in mice. As a result, the carcinogenic risk of styrene has been questioned throughout.

About genotoxicity and carcinogenicity, the effects of working exposure have been investigated for decades; in genotoxicity, positive results in the HPRT gene mutation test for lymphocytes [54], positive results in comet assay and DNA binding test for blood samples, mononuclear leukocytes [55, 56], increasing of the chromosome aberration frequency [57–62], and increasing in lymphocytes micronucleus [61, 63, 64]. In carcinogenicity, incidence of tumours in lymph and hematopoietic, and leukemia [65], incidence increasing of leukemia and lymphoma were observed [66]. On the other hand, some reports said that styrene induced no effect in genotoxicity and carcinogenicity by occupational exposure [67, 68]. There are relatively many incidences reported on positive effects. However, concluding the impact of styrene on human health is difficult because the exposure level was inconcrete, and there is a possibility that people were exposed to not only styrene but also other chemicals in the workplace.

As previously demonstrated, previous studies yielded both positive and negative results for its genotoxicity, implying that research data is still insufficient to definitively determine its genotoxicity. Nevertheless, styrene and SO have been focused and examined for long, there is ambiguity in the previous data collection, and there is inadequate evidence to evaluate the genotoxicity and mode of action. In particular, in gene mutation assays, one of the important hazard identifications, no valid in vivo data were identified in contrast to the case of in vitro test results, the amount of data was available. From previous in vitro data, it can be interpreted that unmetabolized styrene does not have mutagenicity, but SO obviously shows the positive effect in Ames tests and MLA. However, there is no compelling evidence that styrene is carcinogenic in humans. Using MutaMouse, we performed an in vivo gene mutation assay of styrene in the carcinogenic organs of the liver and lung. The goal of this study is to look into the mutagenicity of styrene through oral exposure.

## Materials and methods

The research was carried out in accordance with the OECD Guidelines for Chemical Testing 488. (26 June 2020: Transgenic Rodent Somatic and Germ Cell Gene Mutation Assays). The test was carried out at the BioSafety Research Center (BSRC: Shizuoka, Japan). In accordance with “the Act on Animal Welfare and Management,” “the standards relating to the care and management of laboratory animals and pain relief.” and “BSRC Guidelines for Animal Experimentation.” The animals were cared for in accordance with the “Act on the Conservation and Sustainable Use of Biological Diversity through Regulations on the Use of Living Modified Organisms,” and the “BSRC Safety Management Regulations for Recombinant DNA Experiment.”

### Chemicals

Tokyo Chemical Industry Co., Ltd. (Tokyo) supplied styrene (CAS: 100-42-5, Lot no. MD67J, purity: 100.0%). N-ethyl-N-nitrosourea (ENU), a positive control substance, was purchased from Toronto Research Chemicals Inc. (Ontario, Canada). Corn oil was purchased as a negative control substance from FUJIFILM Wako Pure Chemical Corp. (Osaka).

### Animals and treatment

Male and female CD2F1 mice, as well as a male MutaMouse, were obtained from Japan SLC, Inc. (Shizuoka, Japan) and Transgenic Inc. (Kobe, Japan), respectively. After an 8-day acclimation period, 12 male and female animals were found to be in good health were used in the dose-finding study, and 32 male animals were similarly selected for use in the main study. In the dose-finding study, on both male and female, 3 animals for each dose were treated. As there were no differences in toxicity induced by the oral administration of styrene between males and females in the dose-finding study, only male individuals were treated in the main study. These animals were reared on a basal diet, CRF-1 (Oriental yeast), and water ad libitum. Animals were maintained at a room temperature of 20 °C to 26 °C, relative humidity of 35% to 70%, 12 h light/dark cycle, and 12 air changes per hour. Groups of three CD2F1 mice/sex were administered styrene by gavage once a day for two weeks for dose-finding study at a volume of 10 mL/kg, and at levels of 30.0, 100, 300, and 1000 mg/kg. The highest dose level was set based on the OECD Guidelines for the Testing of Chemicals 488 and the total four levels were divided by geometric ratio 3. Based on the results of the dose-finding study (shown in 3.1), we considered doses of 75, 150, and 300 mg/kg/day to be used for 28-day repeated administration. For the main study, separate groups of the vehicle control (corn oil) were maintained in the same manner. The positive control was treated with ENU (i.p.) at 100 mg/kg/day once a day for 2 days. Euthanasia of experimental animals and the extraction of target organs were conducted 3 days after the last administration in every treatment and negative control group, and 10 days after that in the positive control group. Six animals were treated in all groups (only in the case of 300 mg/kg/day group, 8 animals were treated). Animals were observed once a day every day. Body weight was recorded on the administration days 1, 8, 15, and 22, and one and three day(s) after the last treatment. We selected each of the five animal samples in the ascending order of animal ID for mutation analysis, except for the case of the 75 mg/kg/day dose group, in which an extremely high MF value of animal ID 3103 was appeared, and animal ID 3106 was additionally evaluated.

The liver and lung were collected after the euthanasia using carbon dioxide gas, and a gross pathology examination was conducted. In the liver, two points of the left lateral lobe were hollowed out and were frozen by liquid N_2_ (LN_2_) in each microtube. Leftover lobes and another lobe packed in a plastic bag were crushed and frozen by a flat-bottomed metal container filled with LN_2_. Similarly, the flat-bottomed metal container with LN_2_ crushed and frozen both the left and right lung packed in a plastic bag. Frozen samples were stored in an ultra-deep freezer (set temperature: − 80 °C; standard value: − 90 to − 60 °C) until analysis. Tissues from five animals were analyzed in each group of mutation assay, and when an outlier appeared, one animal was also analyzed.

### DNA isolation

The following procedures were used to extract genomic DNA from the liver and lung [69]. Frozen tissue was homogenized by a pestle with the Dounce buffer (1.7 g N_2_HPO_4_, 0.25 g KH_2_PO_4_, 8.0 g NaCl, 0.20 g KCl, and 20 mL of 0.5 mol/L EDTA in 1000 mL water) in a Dounce homogenizer. EDTA was purchased from NIPPON GENE Co., Ltd. (Tokyo, Japan). The homogeneous mixture was poured into an ice-cold centrifuge tube containing a 0.5 mol/L sucrose (KANTO CHEMICAL Co., Inc. (Tokyo)) in Dounce buffer. After centrifugation at 3000 r/min (1750 G) for 10 min, the supernatant was removed, and the precipitated nuclei/cells were suspended with 3 mL of RNase (prepared by 100 mL of Dounce buffer and 2.0 mL of RNase A (10 mg/mL; NIPPON GENE Co., Ltd.)) and mixed with 3 mL of proteinase K solution (prepared with 200 mg proteinase K (FUJIFILM Wako Pure Chemical Co., Ltd.), 60 mL of distilled water, 20 mL of 10 w/v% SDS solution (SDS: FUJIFILM Wako Pure Chemical Co., Ltd.), and 20 mL of 0.5 mol/L EDTA adjusted to pH 7.5), followed by incubated at 50 °C for 2–2.5 h. A (1:1) mixture of phenol and chloroform was added, and the water layer was separated after 10 min of centrifugation at 2500 r/min (1220 G). Chloroform/isoamyl alcohol (24:1) and the water layer were mixed and centrifuged in the same manner. The water layer was added into another centrifuge tube, and ethanol was added to precipitate the DNA. The DNA was soaked in 70% ethanol for 10 min. After ethanol evaporation, the DNA was dissolved in TE buffer (NIPPON GENE) at room temperature overnight. The DNA solution was placed in the refrigerator at 4 °C. NanoDrop (AGC TECHNO GLASS Co., Ltd. (Shizuoka)) was used to determine the concentration of DNA.

### In vitro packaging

Lambda in vitro packaging reaction was performed for transgene rescue according to the Transpack instruction manual (Agilent Technologies, Transpack Packaging Extract Catalog #200,220, #200,221, and #200,223). Approximately 10 µL of the genomic DNA solution (100—600 μg/mL) was gently mixed with the dedicated Transpack packaging tube and incubated at 30 °C for 1.5 h twice before being mixed with 700 µL of SM buffer that was prepared as follows: 5.84 g NaCl, 2.03 g MgSO_4_·7H_2_O, 50.0 mL of 1 mol/L Tris–HCl [pH 7.5] (NIPPON GENE Co., Ltd.), and 100 mg gelatin powder (KANTO CHEMICAL Co., Inc.) were mixed in 800 mL of ultrapure water and then autoclaved (121 °C, 20 min), after fixing the 1000 mL volume by ultrapure water.

### Mutant frequency determination

A mixture of 2 mL of Escherichia coli C strain (lacZ^−^, gal E^−^) suspension and the whole amount of packaged sample, ~ 700 µL volume, were stirred and incubated for 30 min, and the rescued phages were absorbed into E. coli. This solution was diluted 10 times by adding 30 µL solution into 270 µL of LB culture medium containing 10 mmol/L of magnesium sulfate, and then 30 µL of this dilution was mixed with the E. coli suspension in the titer tube. An aliquot of this suspension was mixed with LB top agar for the titer plates. The remaining cell suspension was mixed with LB top agar containing P-gal (phenyl-β-d-galactoside) for the selection plates. These both plates were incubated overnight at 37 °C. These packaging procedures were repeated until a total of 300,000 plaques were produced. The total number of plaques (N) was calculated by the following formula, using the total number of plaques on the titer plate (n).

$$\mathrm{N}=\frac{\mathrm{n}\times 300 \left(\mathrm{\mu L}\right)\times 2700 \left(\mathrm{\mu L}\right)}{30 \left(\mathrm{\mu L}\right)\times 30 \left(\mathrm{\mu L}\right)}$$

The mutant frequency (MF) was calculated as follows: MF = total number of plaques on selection plates (s) divided by the total number of plaques (N).

$$\mathrm{MF}=\frac{\mathrm{s}}{\mathrm{N}}$$

### Statistical analysis

Bartlett’s test was used to assess data homogeneity in the treatment and negative control groups. When the homogeneity was detected, the Dunnett’s test was used to analyze the data. For non-homogenous data, Steel’s test was used. Based on the result of F-test, the Student’s t-test or Aspin-Welch's t-test was used to compare MFs between negative and positive controls. The criterion for significance was set at 5% levels of probability.

## Results

### Dose-finding test

Styrene-treated males in the 1000 mg/kg/day group demonstrated a decrease in locomotor activity (2/3), irregular respiration (3/3), and prone position (1/3), and were deemed moribund and were euthanized on the Day 2 or 3. Styrene-treated females in the 1000 mg/kg/day group also had a decrease in locomotor activity (2/3), irregular respiration (3/3), prone position (1/3), pale skin (whole body) (3/3), and hypothermia (2/3), and they were euthanized on Day 2 or Day 3. There was no variation in condition or weight in the styrene treatment groups of 30, 100, and 300 mg/kg/day. As a result, the experimental doses in the gene mutation assay were set as 75, 150, and 300 mg/kg/day.

### Main experiment for mutation assay

#### Observation of conditions and body weight

**Fig. 1:**
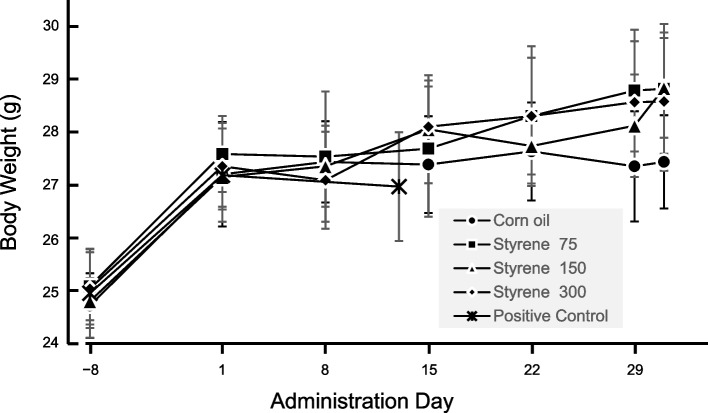
MutaMouse body weight over a 28-day administration period. Each graph depicts the average weight value ± standard deviations (6 animals in negative control, styrene 75 mg/kg/day, styrene 150 mg/kg/day treatment, and positive control groups, 8 animals in styrene 300 mg/kg/day treatment group.)

All styrene-treated groups did not show variation in the general conditions in the main experiment for the mutation assay. Body weights in the treatment groups did not differ significantly from those in the control group (Fig. 1).

### Gross pathological examination

One individual [Animal ID No. 3103] in the styrene treatment (75 mg/kg/day) group had a white patch in the liver and a reddish patch in the lung. One-sixth of the animals in the styrene (150 mg/kg/day) treatment group developed a nodule in the liver. Furthermore, 3/8 of the animals in the styrene treatment (300 mg/kg/day) group showed liver darkening.

### Mutation assay

#### LacZ MFs in the liver

The average value ± standard division of lacZ MFs was 34.1 ± 9.4 (× 10^−6^) in negative control group, whereas in styrene-treated groups, the MFs were 98.5 ± 152.7 (× 10^−6^), 48.7 ± 25.9 (× 10^−6^), and 49.0 ± 11.1 (× 10^−6^) in 75 (1 individual was additionally assayed [Animal ID No. 3106]), 150, and 300 mg/kg/day treatment group, respectively (Table 1). These styrene-treated values were not significantly different from the values in the negative control group (75 mg/kg/day treatment group vs negative control: *p* = 0.81, 150 mg/kg/day treatment group vs negative control: *p* = 0.81, 300 mg/kg/day treatment group vs negative control: *p* = 0.12, Steel’s test). In the 75 mg/kg/day treatment group, the MFs except for the outlier animal [Animal ID No. 3103] were 36.3 ± 10.3 × 10^−6^, and a significant difference was also not observed (75 mg/kg/day treatment group vs negative control: *p* = 0.99, 150 mg/kg/day treatment group vs negative control: *p* = 0.35, 300 mg/kg/day treatment group vs negative control: *p* = 0.34, Dunnett’s test). These MFs were not dose dependent and within the range of historical negative control data (mean ± S.D. = 42.0 ± 12.8, Supplementary table).

**Table 1:** Mutant frequencies in the livers of MutaMouse given styrene [Male mice were dosed once daily for 28 days (Oral administration, tissues were collected 3 days after the final administration)]

Substance	Dose (mg/kg/day, p.o.)	Animal ID No	Number of plaque	Number of packaging	Number of mutants	Mutant frequency (× 10^−6^)	Group Mean ± S.D. (× 10^−6^)
Corn oil	0	3001	310,500	1	13	41.9	34.1 ± 9.4
		3002	306,900	1	8	26.1	
		3003	540,900	1	12	22.2	
		3004	585,900	1	25	42.7	
		3005	587,700	1	22	37.4	
Styrene	75	3101	414,000	1	13	31.4	98.5 ± 152.7 (36.3 ± 10.3) #
		3102	847,800	1	31	36.6	
		3103	798,300	1	327	409.6	
		3104	712,800	1	32	44.9	
		3105	598,500	1	13	21.7	
		3106	956,700	2	45	47.0	
	150	3201	662,400	1	41	61.9	48.7 ± 25.9
		3202	398,700	1	15	37.6	
		3203	386,100	1	10	25.9	
		3204	361,800	1	11	30.4	
		3205	546,300	1	48	87.9	
	300	3301	538,200	1	28	52.0	49.0 ± 11.1
		3302	602,100	1	27	44.8	
		3303	538,200	1	20	37.2	
		3304	736,200	1	49	66.6	
		3305	450,900	1	20	44.4	
ENU	100	3401	489,600	1	58	118.5	109.1 ± 17.1 *
		3402	666,000	1	62	93.1	
		3403	699,300	1	91	130.1	
		3404	666,000	1	60	90.1	
		3405	678,600	1	77	113.5	

Corn oil: Control group (10 mL/kg)

ENU: Positive control (N-ethyl-N-nitrosourea, 10 mL/kg, i.p., once daily for 2 days, expression period; 10 days)

^*^Statistically significant difference from the negative control (Student’s t-test: *p* < 0.05)

^#^This value was obtained by subtracting Animal ID No.3103

The average of MFs in the positive control group was 109.1 ± 17.1 (× 10^−6^), indicating a significant increase from the negative control group (*p* = 0.0001, Student’s t-test.).

### LacZ MFs in the lung

The average value ± standard division of lacZ MFs was 33.4 ± 9.7 (× 10^−6^) in the negative control group, whereas in styrene-treated groups, the MFs were 108.6 ± 130.5 (× 10^−6^), 46.1 ± 20.1 (× 10^−6^), and 43.7 ± 6.1 (× 10^−6^) in 75 (one individual was additionally assayed [Animal ID No. 3106]), 150, and 300 mg/kg/day treatment group, respectively (Table 2). The value in 75 mg/kg/day of styrene treatment was significantly different from the values in the negative control group statistically (75 mg/kg/day treatment group vs negative control: *p* = 0.05, 150 mg/kg/day treatment group vs negative control: *p* = 0.53, 300 mg/kg/day treatment group vs negative control: *p* = 0.28, Steel’s test.). Additionally, the MFs except for the outlier animal [Animal ID No. 3103] were 55.6 ± 16.4 × 10^−6^, and significant difference was not observed from negative control value (75 mg/kg/day treatment group vs negative control: *p* = 0.06, 150 mg/kg/day treatment group vs negative control: *p* = 0.38, 300 mg/kg/day treatment group vs negative control: *p* = 0.54, Dunnett’s test). These MFs were not dose dependent and within the range of historical negative control data (mean ± S.D. = 47.6 ± 14.1, Supplementary table).

**Table 2:** Mutant frequencies in the lungs of MutaMouse given styrene [Male mice were dosed once daily for 28 days (Oral administration, tissues were collected 3 days after the final administration)]

Substance	Dose (mg/kg/day, p.o.)	Animal ID No	Number of plaque	Number of packaging	Number of mutants	Mutant frequency (10^−6^)	Group Mean ± S.D. (× 10^−6^)
Corn oil	0	3001	313,200	1	7	22.3	33.4 ± 9.7
		3002	396,000	1	10	25.3	
		3003	620,100	1	25	40.3	
		3004	383,400	1	13	33.9	
		3005	486,000	1	22	45.3	
Styrene	75	3101	569,700	1	27	47.4	108.6 ± 130.5 * (55.6 ± 16.4) #
		3102	899,100	1	45	50.1	
		3103	830,700	1	310	373.2	
		3104	680,400	1	51	75.0	
		3105	531,000	1	19	35.8	
		3106	543,600	1	38	69.9	
	150	3201	620,100	1	34	54.8	46.1 ± 20.1
		3202	368,100	1	27	73.3	
		3203	854,100	1	25	29.3	
		3204	522,000	1	26	49.8	
		3205	594,900	1	14	23.5	
	300	3301	649,800	1	28	43.1	43.7 ± 6.1
		3302	547,200	1	21	38.4	
		3303	537,300	1	20	37.2	
		3304	927,000	1	47	50.7	
		3305	713,700	1	35	49.0	
ENU	100	3401	737,100	1	123	166.9	180.7 ± 35.0 **
		3402	482,400	1	61	126.5	
		3403	670,500	1	130	193.9	
		3404	763,200	1	153	200.5	
		3405	575,100	1	124	215.6	

Corn oil: Control group (10 mL/kg)

ENU: Positive control (N-ethyl-N-nitrosourea, 10 mL/kg, i.p., once daily for 2 days, expression period: 10 days)

^*^Significant difference from the negative control (Steel's test: *p* < 0.05)

^**^Significant difference from the negative control (Aspin-Welch’s t-test: *p* < 0.05)

^#^This value was obtained by subtracting Animal ID No.3103

The average of the MFs in the positive control group was 180.7 ± 35.0 (× 10^−6^), indicating a significant increase from the negative control group (*p* = 0.0004, Aspin-Welch's t-test.).

## Discussion

There were no deaths in mice treated with styrene up to the highest dose in the main study. The administration of styrene had no effect on the overall conditions or weight changes. However, the presence of gross pathological changes in the liver suggests that styrene was absorbed and reached the target organs. The treated groups did not show a significant increase in MFs in the liver of styrene-treated MutaMouse. Except for the 75 mg/kg/day dose, the mutations in the lung were not increase in the treated groups. The increase showed no dose-dependency, and only one animal in this group showed very high MF (373.2 × 10^−6^) exceeding the background control. Therefore, that was considered to be an accidental clonal mutation (the value shown in table was also calculated by removing this animal). Although the MF value of negative control was slightly lower than those of the treated groups, there was no significant difference and the MFs were within the range of historical negative control data (Supplementary table). Consequently, the mutagenicity of styrene was determined to be negative in the liver and lung of MutaMouse in this experimental condition.

Although 1000 mg/kg/day has been shown to exceed the MTD in the dose-finding study, at the highest dose in the main experiment, 300 mg/kg/day, neither weight loss nor clinical symptoms were observed, except for the gross pathological findings in the liver. This suggests a concern regarding the negative result due to an insufficient maximum dose causing the low amount of SO produced by the styrene metabolism. However, the oral dose of 300 mg/kg/day was carcinogenic in mouse lungs [47]. In a dose-finding experiment for the abovementioned carcinogenicity study (5 days/week for 7-week administration) [47], high mortality was observed at 681 mg/kg (486 mg/kg/day), and body weight gain was decreased at 316 mg/kg (226 mg/kg/day).

Traditionally, liver and stomach tissues are frequently sampled for verification in gene mutation assays using transgenic rodents, taking into account the results of liver and stomach with corresponding to metabolic functional and direct-contact tissues. In this study, however, liver and lung tissues were used as samples. It had been previously reported that the lung cancer occurred for two routes of exposure, gavage and inhalation, in male and female O20 mice [49], in male B6C3F mice [48], and in male and female CD-1 mice [50]. Thereby, the mutagenicity in the lungs was especially verified. In addition, DNA adducts were found in several tissues such as the liver and lung in rodent studies [54, 70–73]. Regarding other in vivo assays, the comet assay in the liver tissue of mouse showed positive results [74, 75]. Our findings show that mutagenic effects were not observed in the lungs of mice that gavaged styrene at a carcinogenicity level [48] of 300 mg/kg/day.

In the metabolites of styrene, the most toxic and the highest reactive substance is SO [76]. The generation of SO is a possible process to state the mutagenesis and carcinogenesis of styrene. The genotoxicity of SO and its inducing tumor have already been proved by some experiments [14, 24, 51, 77, 78]. Previous data showed significant differences in the appearance of the toxic effects of styrene in vitro and in vivo. In vitro data, such as Ames test results, clearly show the mutagenic positive through the SO existence, whereas in vivo data evaluating styrene has consistently shown the negative result, including this study.

Some studies represent the evidence of SO production and DNA adduct formation induced by inhalation exposure of styrene in vivo [54, 71, 79]. However, it does not mean that these adducts result in the induction of gene mutations. In contrast, the in vivo mutagenicity of orally administered styrene was found to be negative in our study. Furthermore, CA in the bone marrow of orally-treated mice yielded a negative result [46]. It may suggest that SO is possibly metabolized and decomposed immediately within organisms. The investigation of the production and distribution of SO and SO-induced DNA adducts will be a significant step in unravel the mechanism of carcinogenicity of styrene and the explanation of diremption between the in vivo negative and the in vitro positive mutagenic results.

The physiological pharmacokinetic model study reported that the efficiency of styrene metabolism saturates at 200 ppm inhaled exposure concentration in mice, rats, and humans [6]. The metabolic capacity of styrene in the liver is different among species. The generative capacity of SO, which means the activity of NADPH-cytochrome P450 monooxygenase, is higher in mice than in rats, that of humans follows these [80]. Moreover, particularly in humans, the capacity of metabolism from SO into styrene glycol is highest among them [80]. These species differences could be taken into consideration when the human health risks are assessed on the base of animal experiment; If in vivo mutagenic negative result in this study is attributed to the SO decomposition by metabolizing ability, the human health risk of styrene could be probably lower than the estimation from the animal experiment. In the case that in vivo mutagenicity of styrene is considered as negative and there is no involvement of SO, the styrene might be potentially a non-genotoxic carcinogen.

In particular, in mice, a distinctive hypothetical mode of action was reported [81]. The specific enzyme, CYP2F2 exert its oxidative action and leads to the form of ring-oxidized metabolites of styrene in lung club cell. These metabolites have been implicated in the physiological impact other than the gene mutation; changes in gene expression for lipid and lipoprotein metabolism, cell cycle, and mitotic M-M/G1 phase; cytotoxicity and mitogenesis in club cells; disruption of the circadian cycle; and progression to preneoplastic/neoplastic lesions in the lungs. These physiological effects, rather than the genotoxic effects of SO, may be responsible for the development of lung cancer. In human, there is no report showing this carcinogenic mechanism as far as we know, and this mode of action is specific in mice [81, 82]. Moreover, as a recent study, the significant association of the aldehyde dehydrogenase 2 (ALDH2) in the styrene metabolism was revealed by the epidemiological survey and therefore, it was suggested that the carcinogenicity induced by the occupational exposure to styrene could be particularly modified by ALDH2 polymorphisms [83]. More detailed information and research findings are still needed to clear up the styrene reaction in humans.

IARC referred to the previous data and classified the styrene as the carcinogenicity classification “Group 2A” [47]. Currently, many regulatory bodies divide carcinogens as mutagenic carcinogens or non-mutagenic carcinogens for their risk assessment [84]. For example, ICH guideline M7 shows the acceptable intake (AI) of styrene as 154 µg/day based on the calculation for a mouse inhalation study [50, 85] treating styrene as a mutagenic carcinogen. Hence, the negative result in the gene mutation assay in the present study can be a remarkable finding. The result could suggest that styrene is not mutagenic with oral exposure.

In recent, the updating risk assessment by IARC [47], the genotoxic review by Moore et al., (2019) [10] and the meta-analysis in micronuclei of epidemiologic studies [86], etc. are the valid information concerning styrene genotoxicity; nevertheless both reports are suggesting that there is no conclusive evidence whether styrene induces the mutagenicity in vivo via the metabolism to styrene oxide. Besides, the scientific opinion was also released by the European Food Safety Authority (EFSA), saying that the concern of genotoxicity associated with oral exposure to styrene couldn’t be excluded [87]. Moreover, there has been an increasing concern today over microplastic pollution in oceans and food chains. SO is a common plasticizing compounds and causes DNA damage and mutagenesis in human cells in vitro [88]. Therefore, in terms of the global public health, as well, it is extremely important to clarify the health impact and the mechanism of action of styrene. Scientific data and evidence were so insufficient that we could not determine the genotoxicity and carcinogenicity of styrene as a risk of human health. Hence, this study suggesting the negative result in gene mutation assay with oral exposure can be a notable finding. The result could suggest that styrene is not mutagenic in vivo. For better understanding of the toxicity by styrene exposure and risk control in the society, it is necessary to investigate the mechanism of genotoxicity and carcinogenicity by styrene in more details.

## Supplementary Information

**Additional file 1. **Supporting information table 1 data from previous controls (transgenic rodent gene mutation assay (*lacz* assay)

## Data Availability

All available data are shown in this article.
