# Supplementary material for: In vivo mutagenicity assessment of styrene in MutaMouse liver and lung
Source: Genes Environ. 2023 Apr 11;45:12. doi: 10.1186/s41021-023-00270-9 (PMC10088199; doi:10.1186/s41021-023-00270-9)
Supplement: Supplementary file 1 — Additional file 1. Supporting information table 1 data from previous controls (transgenic rodent gene mutation assay (lacz assay) [file 41021_2023_270_MOESM1_ESM.docx]

Supporting Information Table 1 Data from previous controls (Transgenic rodent gene mutation assay (*lacZ* assay))

| Group | n | Mutant frequency [×10^−6^] (Mean ± S.D.) | Acceptable range * | |
| --- | --- | --- | --- | --- |
|  |  |  | Lower | Upper |
| [MutaMouse: male; liver] Negative control | 122 | 42.0 ± 12.8 | 16.9 | 67.1 |
| [MutaMouse: male; lung] Negative control | 25 | 47.6 ± 14.1 | 20.0 | 75.2 |

Liver: The historical control data presented above was compiled from April 19, 2017 to December 18, 2020.

Lung: The historical control data presented above was compiled from June 2, 2004 to April 17, 2019.

Negative control: Water, 0.5% methylcellulose, corn oil, and other ingredients are included.

* The confidence interval for negative control is 95%.
